# Pretreatment Pan-Immune-Inflammation Value Efficiently Predicts Survival Outcomes in Glioblastoma Multiforme Patients Receiving Radiotherapy and Temozolomide

**DOI:** 10.1155/2022/1346094

**Published:** 2022-11-28

**Authors:** Erkan Topkan, Ahmet Kucuk, Ugur Selek

**Affiliations:** ^1^Department of Radiation Oncology, Baskent University Medical Faculty, Adana, Turkey; ^2^Clinic of Radiation Oncology, Mersin Education and Research Hospital, Mersin, Turkey; ^3^Department of Radiation Oncology, Koc University School of Medicine, Istanbul, Turkey; ^4^Division of Radiation Oncology, The University of Texas MD Anderson Cancer Center, Houston, Texas, USA

## Abstract

**Objectives:**

The purpose of this study was to determine the predictive significance of pretreatment pan-immune-inflammation value (PIV) in patients with newly diagnosed glioblastoma multiforme (GBM) who received postsurgical radiation (RT) and concurrent plus adjuvant temozolomide (TMZ).

**Methods:**

The outcomes of 204 newly diagnosed GBM patients were analyzed retrospectively. Each eligible patient's PIV was calculated using the findings of peripheral blood platelet (P), monocyte (M), neutrophil (N), and lymphocyte (L) counts obtained on the first day of therapy: PIV = *P* × *M* × *N* ÷ *L*. We used receiver operating characteristic (ROC) curve analysis to discover the ideal cutoff values for PIV concerning progression-free (PFS) and overall survival (OS) outcomes. The primary and secondary end-points were the OS and PFS divergences across the PIV groups.

**Results:**

In ROC curve analysis, the optimal PIV cutoff was 385, which substantially interacted with PFS and OS results and categorized patients into low PIV (L-PIV; *N* = 75) and high PIV (H-PIV; *N* = 129) groups. Comparative survival analyses showed that the patients in the H-PIV group had significantly shorter median PFS (6.0 vs. 16.6 months; *P* < 0.001) and OS (11.1 vs. 22.9 months; *P* < 0.001) durations than those in the L-PIV group. The results of multivariate Cox regression analysis indicated an independent and significant connection between an H-PIV measure and shorter PFS and OS outcomes.

**Conclusions:**

The novel PIV was able to independently stratify newly diagnosed GBM patients into two groups with fundamentally different PFS and OS outcomes following RT and concurrent plus adjuvant TMZ.

## 1. Introduction

Glioblastoma multiforme (GBM) is the most common adult glial tumor, accounting for one-third of all primary brain tumors [1]. Although maximal safe resection followed by radiotherapy (RT) plus concurrent and adjuvant temozolomide (TMZ) approach (Stupp regimen) yields the best results, the prognosis of such patients is bleak, with an estimated 5-year survival rate of less than 10% [2, 3]. Regrettably, neither breakthroughs in imaging, surgical, and RT techniques nor chemotherapy seemed to improve survival rates beyond those obtained with the standard Stupp regimen [4, 5]. Tumor-treating fields (TTF) therapy was authorized as a novel therapeutic strategy for newly diagnosed GBM patients, as its concurrent use with adjuvant TMZ resulted in a substantial survival benefit over standard adjuvant TMZ alone [6]. However, even with TTF, the study's median overall survival (OS) rate was only 20.9 months, reflecting the drama of such patients.

The widely recognized prognostic factors for newly diagnosed GBM include the patients' age, Eastern Cooperative Oncology Group (ECOG) performance and neurologic function status, presence/absence of increased intracranial pressure, recursive partitioning analysis (RPA) group, the extent of resection, need for steroids, chemoradiotherapy scheme, adjuvant chemotherapy choice, and the presence/absence of the genetic and molecular markers like isocitrate dehydrogenase 1/2 (IDH-1/2) mutation, 1p/19q codeletion, and O6-methylguanine-DNA methyl-transferase (MGMT) gene promoter methylation [7, 8]. These characteristics, alone or in combination, resulted in the successful stratification of such patients. However, there are hard-to-explain discrepancies in the ultimate survival results of patients with indiscernible clinical, pathological, genetic, molecular, and therapeutic aspects, underscoring the critical need for innovative prognosticators with higher prognostic strengths.

There is substantial evidence that systemic inflammation contributes mightily to the development of gliomas, the progression of the disease, and the prognosis of patients treated with comparable therapies [9]. Several blood-borne indicators of systemic inflammation, including cellular components or serum proteins like platelets, monocytes, neutrophils, lymphocytes, C-reactive protein, and albumin, have been examined for their prognostic usefulness in GBM patients. The findings of such research invariably indicated a robust relationship between the survival results of GBM patients and these biomarkers, either individually or in unique combinations [10–18]. The pan-immune-inflammatory value (PIV) is a newly created immune inflammation measure that is a unique combination of monocyte, platelet, neutrophil, and lymphocyte counts [19]. In previous reports, PIV showed a strong association with OS in patients with advanced colorectal cancer, advanced breast cancer, esophageal cancer, small-cell and non-small cell lung cancers, and Merkel cell carcinoma who underwent surgery and/or systemic therapy [19–28]. Gliomas, particularly GBM, have a severe inflammatory and immunosuppressive milieu that permits them to evade the antitumor immune response, evidencing that the novel PIV might be employed as a likely predictor of outcomes in such patients [9]. As a result of the unavailability of GBM research, we conducted this retrospective cohort study to explore the possible prognostic utility of PIV in newly diagnosed GBM patients who underwent the standard Stupp regimen.

## 2. Patients and Methods

### 2.1. Study Population

We retrospectively reviewed the medical records of all newly diagnosed GBM patients who underwent postoperative RT plus concurrent and adjuvant TMZ between February 2007 and December 2020 at Baskent University Medical Faculty Department of Radiation Oncology. Patients fitting the following requisites were eligible for the study: aged 18 to 80 years, ECOG of 0-1, histologically confirmed GBM diagnosis according to WHO classification, no prior chemotherapy or cranial RT, available preoperative and postoperative gadolinium-enhanced magnetic resonance imaging (MRI) scans, available chemotherapy and RT details, existing pretreatment complete blood count and biochemistry tests with adequate hematologic, renal, and hepatic functions, no direct evidence of active infection, and no prior immunosuppressive disease history. The use of nonstandard RT methods such as whole-brain RT or hypofractionated RT, as well as the absence of TMZ administration during either the concurrent or adjuvant treatment phases, were all exclusion criteria.

### 2.2. Ethics, Consents, and Permissions

The current study was carried out following the postulates of the Helsinki Declaration and its successors, and its methodology was approved by the institutional review board before any patient data was collected. Before commencing the prescribed therapy, each qualifying patient signed an informed consent form authorizing the collection and analysis of blood samples, pathologic specimens, and academic publication of their findings by themselves or lawfully commissioned deputies.

### 2.3. Treatment Protocol

All patients were first assessed and underwent maximal safe resection if deemed practicable, as instructed by our institutional norms for GBMs. A total dose of 60 Gy (2.0 Gy/fx, for 30 days) of partial brain RT was delivered after surgery using 3-dimensional conformal (3D-CRT) or intensity-modulated RT (IMRT). According to our corporate care standards, all treatment plans were carried out using coregistered CT and contrast-enhanced MRI fusion images irrespective of the RT technique used. During the whole course of RT, concurrent TMZ (75 mg/m^2^/day, once daily, seven days a week, for six weeks) and prophylactic trimethoprim-sulfamethoxazole against Pneumocystis jirovecii were administered to all patients. Adjuvant 6 to 12 cycles of TMZ (150/200 mg/m^2^/day, for 5 days, every 28 days) were prescribed for all patients starting at 3 to 4 weeks of completion of RT and TMZ. Additional medications, like antiepileptic drugs, were utilized if only clinically indicated.

### 2.4. Measurement of PIV

The PIV was calculated as *P* × *M* × *N* ÷ *L*, where *P*, *M*, *N*, and *L* represent pretreatment platelet, monocyte, neutrophil, and lymphocyte counts acquired on the first day of concurrent RT and TMZ [19].

### 2.5. Response Assessment

We used brain MRI scans acquired at 2- and 3-month intervals for the first and second follow-up years and every 6 months or more frequently as needed thereafter, following the recommendations of the Response Assessment in Neuro-Oncology (RANO) working group report to evaluate therapeutic response [29]. The records indicated the best response achieved at any time point following the completion of the RT and concurrent TMZ.

### 2.6. Statistical Analyses

The primary endpoint was determined as the potential link between the pretreatment PIV values and the overall survival (OS) results, defined as the interval between the initiation of RT plus concurrent TMZ and the date of death/last visit. The secondary endpoint was the progression-free survival (PFS: the time interval between the initiation of RT plus concurrent TMZ and the date of the first observation of disease progression or death/last visit). Medians and ranges were employed to express quantitative variables, while categorical variables were described as percentage frequency distributions. The Pearson *χ*^2^ test was used to compare demographic characteristics between groups. The research participants were separated into the requisite number of groups for intergroup comparisons if necessary. To estimate survival results, Kaplan-Meier survival curves were operated, with two-sided log-rank test analyses being employed for intergroup comparisons. For multivariate comparisons, the Cox proportional hazards model was applied, with those factors that indicated significance in univariate comparisons included. Any 2-tailed *P* < 0.05 was deemed statistically significant.

## 3. Results

Table 1 summarizes the pretreatment patient and disease features of all 204 patients who participated in the investigation. The committed RT dosage and concurrent TMZ were administered to all patients.

Of all the eligible patients, 19 (9.3%) were still alive and 14 (6.9%) remained progression-free after a median follow-up time of 17.6 months (range = 2.4–108.3). The overall study cohort's median PFS and OS times were 10.3 months (95% confidence interval (CI): 7.8–13.1 months) and 15.8 months (95% CI: 13.0–18.6 months), respectively. The matching 5-year PFS, OS, and actuarial brain control rates were 6.4%, 7.3%, and 6.9%, respectively. In the absence of extracranial metastases, the most common treatment failure patterns were infield (≥80% of T1 enhanced tumor volume was within 95% isodose line) and marginal (>20% but ≤80% of the tumor volume was within the 95% isodose line), which accounted for 83.5% and 8.2% of all cases, respectively.

**Figure 1:**
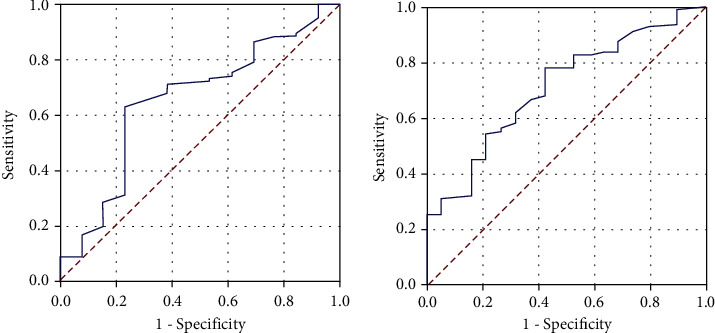
The results of receiver operating characteristic curve analyses. (a) Progression-free survival. (b) Overall survival.

**Figure 2:**
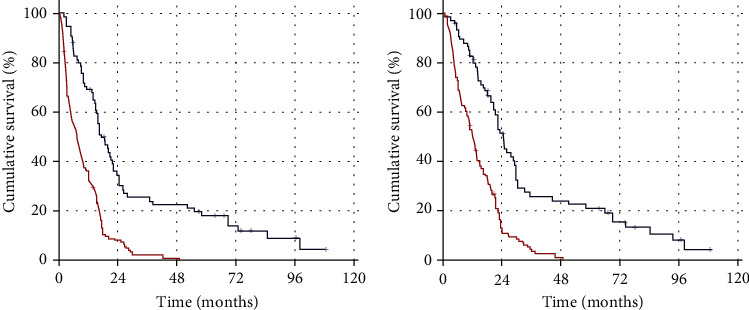
Comparative survival outcomes between the pan-immune-inflammation value (PIV) groups. (a) Progression-free survival. (b) Overall survival (dark blue: low pan-immune-inflammation value; red: high pan-immune-inflammation value).

**Figure 3:**
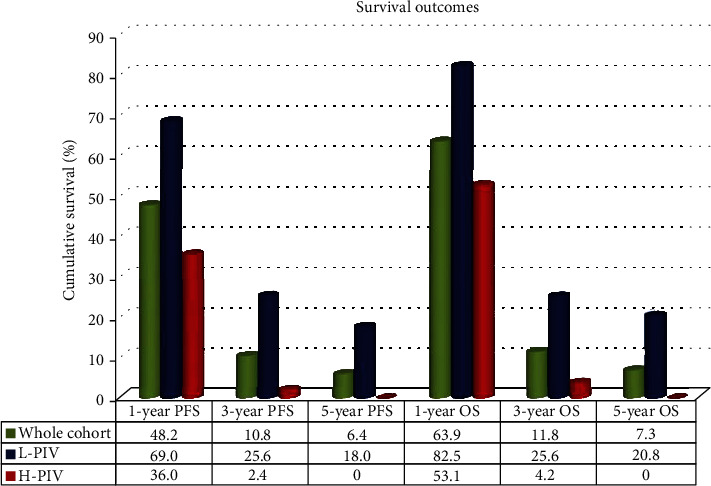
The bar chart demonstrating the 1-, 3-, and 5-year survival outcomes for the entire study population and per pan-immune-inflammation value (PIV) groups (PFS: progression-free survival; OS: overall survival; L-PIV: low pan-immune-inflammation value; H-PIV: high pan-immune-inflammation value).

Receiver operating characteristic curve analysis determined the ideal PIV cutoffs as 382 (area under the curve (AUC): 69.7%; sensitivity: 67.4%; specificity: 65.6%) for PFS and 388 (AUC: 72.7%; sensitivity: 68.2%; specificity: 66.4%) for OS, which display significant connections with the results (Figure 1). However, because both cutoffs were numerically close, the research cohort was divided into two groups with rounded cutoff values of 385 (arithmetic average of two values) for intergroup comparisons: low PIV (L-PIV): PIV < 385 (*N* = 75) and high PIV (H-PIV): PIV ≥ 385 (*N* = 129). Baseline demographic comparisons indicated that the two PIV groups had nearly identical distributions of all characteristics (Table 1). The therapeutic features of the initial RT and TMZ, as well as adjuvant TMZ and salvage therapies were, identical between the L-PIV and H-PIV groups (Table 2). But, although the failure patterns were similar between the two groups, comparative analysis showed that the H-PIV cohort had a significantly higher total brain failure rate than its L-PIV counterpart (93.1 percent vs. 84 percent, *P* = 0.02) (Table 2). Comparative Kaplan-Meier curve estimates revealed that the H-PIV group had significantly shorter median PFS (7.3 vs. 17.4 months, *P* < 0.001) and OS (12.4 vs. 24.9 months, *P* < 0.001) durations than the L-PIV group (Figure 2). Likewise, the 1-, 3-, and 5-year PFS and OS rates were markedly inferior in the H-PIV group (Table 2 and Figure 3). Of note, there was no 5-year survivor in the H-PIV group compared to 20.8% of the L-PIV group.

Results of the univariate analyses revealed the pretreatment H-PIV group (vs. L-PIV), subtotal resection/biopsy only (vs. gross total resection), and RTOG RPA class V (vs. class III-IV) as the factors related to significantly worse PFS and OS outcomes, all of which maintained their independent significance in multivariate analysis (Table 3).

## 4. Discussion

The present retrospective cohort study sought to determine the prognostic power of novel PIV in newly diagnosed GBM patients treated with standard Stupp regimen. Our findings suggest a clear relationship between the patients' adverse immune-inflammation status and poor clinical outcomes, as a higher PIV (≥385) was associated with significantly worse PFS and OS outcomes independent of the other prognostic variables, namely the well-established tumor resection type and the RTOG RPA classification.

The extent of the surgery, ECOG performance status, and RTOG RPA class comprise the fittest conventional nongenetic prognostic variables in GBM patients undergoing the standard Stupp regimen. However, underpinning the need for more objective stratification methods, these parameters bear the risk of being influenced by the surgeon's neurosurgical expertise and the radiation oncologists' possible subjectivity when recording the patient's performance score component of the RTOG RPA class. Additionally, similar to our multivariate results, Chaichana et al. argued that age, a component of the RTOG RPA classification, was not a valid predictor of outcomes [30]. As a different approach, limited research has shown that blood-borne platelets, monocytes, neutrophils, and lymphocytes, which are ubiquitous in the highly inflamed GBM microenvironment, have high prognostic usefulness either alone or in distinct unique blends [31]. Constructing sound grounds for our current research, despite the facts conclusively confirming its prognostic competence in various extracranial malignancies [19–28], the unique PIV has never been examined for its prognostic potential in GBM patients. In this respect, the present research represents the first effort to investigate the prognostic strength of novel PIV in newly diagnosed GBM patients who underwent the standard Stupp regimen.

The most striking finding of our study was the demonstration of a strong and independent prognostic significance for pretreatment PIV in such patients, with PIV ≥ 385 measures being linked to significantly lower median OS (12.2 vs. 22.9 months; *P* < 0.001) and PFS (10.3 vs. 16.2 months; *P* < 0.001) than their PIV < 385 counterparts. What is more, substantiating the long-term prognostic relevance of high PIV values, none of the PIV ≥ 385 patients could survive beyond 5 years, compared to a 5-year OS rate of 20.8% in the PIV < 385 patients. Similarly, the 5-year PFS rate was also substantially lower in the PIV ≥ 385 cohort (0% vs. 18% for PIV < 385). Given that these results were acquired with using almost indistinguishable salvage regimens, they collectively hint the PIV ≥ 385 GBM as an exceedingly aggressive tumor phenotype that is resistant to both initial standard therapy and salvage therapies. It is challenging to discuss these results in a proof-based manner, as they have no credible predecessors in the GBM literature. Nonetheless, they appear to be in flawless harmony with previously published PIV research for other cancer sites [19–28] as well as one SII and one SIRI study recently reported by our team for GBM patients managed in a similar manner [17, 18]. The findings of our previously published study on the effect of SII in similarly treated GBM patients indicated notably shorter median PFS (6.0 vs. 16.6 months; *P* < 0.001) and OS (11.1 vs. 22.9 months; *P* < 0.001) durations in the high SII than low SII patient cohorts [17]. The second trial, in which we addressed at the prognostic importance of pretreatment SIRI, discovered that the high SIRI group had substantially inferior median PFS (6.6 vs. 16.2 months; *P* < 0.001) and OS (12.2 vs. 22.9 months; *P* < 0.001) than the high SIRI cohort [18]. Moreover, multivariate Cox regression analysis substantiated SII and SIRI as independent predictors of PFS and OS, respectively, in both research. In comparison to SII and SIRI, the innovative PIV is a more comprehensive biological marker since it incorporates both platelet and monocyte counts into its formula simultaneously: PIV = platelet count × SII or PIV = monocyte count × SIRI. Therefore, our present findings make sense given that both platelets and monocytes of PIV are involved in increased cell survival and proliferation, tumor development, worsened chronic local and systemic inflammation, and decreased antitumor immunity.

The poor PFS and OS outcomes found in our H-PIV GBM group may be explained by the presence of increased systemic inflammation in combination with significantly reduced antitumor immunity; however, the exact mechanisms underlying these results remain unknown. A recent meta-analysis by Feng et al. confirmed the critical role of chronic systemic inflammation, the seventh hallmark of cancer, in gliomagenesis, and glioma prognosis, linking elevated circulating levels of pro-inflammatory markers to a significantly increased risk of glioma development and worse prognosis [32]. These findings were bolstered by the discovery that inflammatory and immune cells account for approximately 30% of total GBM mass [33], with the great bulk of them supporting GBM genesis, growth, and invasiveness. Among these cells, elevated neutrophil counts have long been linked to accelerated tumor growth and treatment resistance [34], with GBM exhibiting the highest neutrophil infiltration of all gliomas [35]. Neutrophils may hamper cytolytic CD8+ T-cells, natural killer cells, and CD4+ suppressor T cells, which may aid GBM cell survival and disease progression by generating an immunosuppressive microenvironment [36]. Furthermore, also the GBM itself causes severe exhaustion, accelerated aging, reduced antitumoral functions, and loss of proliferative capacity in T-cells, to a point where senescent T-cells are unable to proliferate even when stimulated [37, 38]. In gliomas, as a source of tumor-associated macrophages, monocytes are recruited to the brain parenchyma [39], where they acquire the tumor-promoting immunosuppressive properties of myeloid-derived suppressor cells after cell-to-cell contact with GBM cells [40]. Platelets and their aggregates may favor tumor progression by facilitating NF-*Κ*b mediated epithelial-mesenchymal transition, protecting tumor cells from immune surveillance via TGF*-β* mediated down regulation of NKG2D on the surface of NK-cells, and direct protection of GBM cells via cloak formation [41–44]. Although it is difficult to establish a direct hypothetical link between the local immune cell infiltrate of GBM and the systemic immune and inflammation response, destroying the cardinal dogma that the central nervous system (CNS) is an immune-privileged site, evidence has demonstrated that the central nervous system (CNS) is not an immune-privileged site since Medawar's groundbreaking discovery in 1948, which was later confirmed by Nedergaard in 2013 [45, 46]. Therefore, such preliminary evidence, when combined with the previously mentioned SII and SIRI studies in GBM patients, suggests that increased levels of proinflammatory and immunosuppressive platelets, monocytes, and neutrophils, as well as decreased levels of anti-inflammatory and immune-competent lymphocytes, may be responsible for the deteriorated PFS and OS results in the high PIV cohort, as we observed here.

One critical topic that has yet to be solved is whether PIV is a reliable prognostic factor. Because it is one replicable and objectively measurable biochemical factor, the novel PIV seems to meet the criteria for being prognostic for newly diagnosed GBM patients: any patient or disease-related feature that is objectively measurable and provides information on the likely outcome of cancer in untreated individuals [47]. Likewise, regardless of the patient's clinical situation, the four cellular components of PIV are available at no additional expense as part of a routine complete blood count assay, are simple to compute, and are relevant to all patients. In our opinion, the novel PIV has the potential to be a reliable and independent prognostic factor for newly diagnosed GBM patients. This opinion is based mostly on its efficient utility in different tumor locations, the aforementioned unique features, and discriminative capacity to stratify patients into discrete groups with significantly varied PFS and OS results, as established in our study. As a result, if ratified, novel PIV could be a valuable addition to traditional prognosticators, allowing for the stratification of GBM patients into two prognostic groups, with individualized treatment selections enhancing one group's outcomes while sparing the other from the hopeless complications of aggressive treatment schemes.

Several factors limit the strength of the current study. First, our research is a single-center retrospective cohort analysis without a validation cohort, which is prone to selection bias, a typical problem in such studies. Second, as we chose only similarly treated individuals with a presenting ECOG performance score of 0-1, the presented results may not be typical of all GBM patients, as some may have poorer performance scores and/or receive different RT or chemotherapy regimens. Third, these findings ought to be appreciated with caution due to variances in salvage therapies that, while statistically insignificant, might have skewed the final results in favor of either PIV group. Fourth, we were unable to perform PIV group specific analysis according to the genetic markers owing to a lack of patient identification and categorization per MGMT methylation, isocitrate dehydrogenase-1 (IDH-1) and IDH-2, PDGF, PTEN, EGFR, p53, ATRX, and TERT status. And fifth, while individual or simultaneous broad variations in the counts of the PIV components may significantly alter the optimal cutoff during the RT plus TMZ and maintenance TMZ periods, our PIV measures and associated cutoff values only reflect the results of a single time point snapshot. Hence, our discoveries should be treated cautiously and accepted as hypothesis-generating rather than firm guides until the results of suitably designed large-scale research become available. Nevertheless, despite such limitations, if our findings are verified, we assume that they will be useful in prognostic stratification of such patients, which would be useful in projecting the patient prognosis and, hopefully, determining the best treatment options with the advent of more efficient anti-GBM medications.

## 5. Conclusions

While more research is needed to substantiate our findings, the current study discovered that the novel comprehensive PIV, an affordable, noninvasive, readily accessible, simple to compute, and reproducible biological marker, was able to independently stratify newly diagnosed GBM patients into two groups with significantly different PFS and OS outcomes after partial brain RT and concurrent TMZ followed by adjuvant TMZ.

## Figures and Tables

**Table 1:** Baseline patient and disease characteristics.

Characteristic	Whole cohort (*n* = 204)	L-PIV (*n* = 75)	H-PIV (*n* = 129)	*P* value
Median age, *y* (range)	58 (21-80)	60 (34-80)	57 (21-79)	0.73
Age group, *n* (%)				
< 50 years	65 (31.9)	24 (32.0)	41 (32.2)	0.85
≥ 50 years	139 (68.1)	51 (68.0)	88 (68.2)	
Gender, *n* (%)				
Female	69 (33.8)	27 (36.0)	42 (32.6)	0.32
Male	135 (66.2)	48 (64.0)	87 (67.4)	
ECOG, *n* (%)				
0	122 (59.8)	44 (58.7)	78 (60.5)	0.71
1	82 (40.2)	31 (41.3)	51 (39.5)	
RTOG RPA class, *n* (%)				
III	79 (38.7)	29 (38.7)	50 (38.8)	0.91
IV	84 (41.1)	31 (41.3)	53 (41.1)	
V	41 (20.2)	15 (20.0)	26 (20.1)	
Median symptom duration, months (range)	2.1 (0.3-6.2)	2.3 (0.3-6.2)	1.9 (0.3- 4.8)	0.43
Symptom duration group, *n* (%)				
< 3 months	148 (72.5)	55 (73.3)	93 (72.1)	0.88
≥ 3 months	56 (27.5)	20 (26.7)	36 (27.9)	
Extent of surgery, *n* (%)				
Gross total	71 (34.8)	25 (33.3)	46 (35.7)	0.69
Subtotal	99 (48.5)	38 (50.7)	61 (47.3)	
Biopsy	34 (16.7)	16 (16.0)	9 (17.0)	
Anticonvulsant use, n (%)				
Yes	76 (37.3)	27 (36.0)	49 (38.0)	0. 48
No	128 (62.7)	48 (64.0)	80 (62.0)	
Corticosteroid use, *n* (%)				
Yes	114 (67.1)	41 (54.7)	73 (56.6)	0.54
No	90 (29.9)	34 (45.3)	56 (33.4)	

Abbreviations: L-PIV: low pan-immune-inflammation value; H-PIV: high pan-immune-inflammation value; ECOG: Eastern Cooperative Oncology Group; RTOG RPA: Radiation Therapy Oncology Group recursive partitioning analysis.

**Table 2:** Treatment characteristics and clinical outcomes.

Characteristic	Whole cohort (*n* = 204)	L-PIV (*n* = 75)	H-PIV (*n* = 129)	*P* value
Adjuvant TMZ cycles, *n* (%)				
1-6	84 (41.2)	33 (44.0)	51 (39.5)	0.32
7-12	120 (58.8)	42 (56.0)	78 (60.5)	
Brain failure, *n* (%)				
Absent	14 (6.9)	12 (16.0)	2 (1.6)	0.02
Present	190 (93.1)	63 (84.0)	127 (98.4)	
Brain failure type, *n* (%)				
None	14 (6.9)	12 (16.0)	2 (1.6)	0.19
Infield	164 (80.4)	56 (74.6)	108 (83.7)	
Marginal	16 (7.7)	5 (6.7)	11 (8.5)	
Distant	4 (1.9)	0 (0)	4 (3.1)	
Infield and distant	5 (2.6)	2 (2.7)	3 (2.3)	
Marginal and distant	1 (0.5)	0 (0)	1 (0.8)	
Salvage treatment, *n* (%)				
Absent	71 (34.8)	27 (36.0)	44 (34.1)	0.91
Present	133 (65.2)	48 (64.0)	85 (65.9)	
Salvage treatment, *n* (%)				
None	71 (34.8)	27 (36.0)	44 (34.1)	0.57
BEVIRI	38 (18.7)	14 (18.7)	24 (18.6)	
RO	17 (8.3)	7 (9.3)	10 (7.8)	
SRS/SRT	17 (8.3)	6 (8.0)	11 (8.5)	
RO + SRS/SRT	16 (7.8)	6 (8.0)	10 (7.8)	
RO + BEVIRI	19 (9.4)	6 (8.0)	13 (10.0)	
RO + SRS + BEVIRI	17 (8.3)	6 (8.0)	11 (8.5)	
Unknown	9 (4.4)	3 (4.0)	6 (4.7)	
Progression-free survival				
Median, mo. (95% CI)	10.3 (7.8-131)	17.4 (13.3-21.4)	7.3 (5.3-9.3)	<0.001
1-year, %	48.2	69.0	36.0	0.007
3-year, %	10.8	25.6	2.4	<0.001
5-year, %	6.4	18.0	0	<0.001
Overall survival				
Median, mo. (95% CI)	15.8 (13.0-18.6)	24.9 (22.0-27.8)	12.4 (10.5-14.2)	<0.001
1-year, %	63.9	82.5	53.1	0.003
3- year, %	11.8	25.6	4.2	<0.001
5-year, %	7.3	20.8	0	<0.001

Abbreviations: L-PIV: low pan-immune-inflammation value; H-PIV: high pan-immune-inflammation value; TMZ: temozolomide; BEVIRI: bevacizumab plus irinotecan; RO: reoperation; SRS/SRT: stereotactic radiosurgery/stereotactic radiotherapy.

**Table 3:** The bar chart demonstrating the 1-, 3-, and 5-year survival outcomes for the entire study population and per pan-immune-inflammation value groups.

Variable	Progression-free survival			Overall survival		
	Univariate *P* value	Multivariate *P* value	HR	Univariate *P* value	Multivariate *P* value	HR
Age (≤50 vs. >50 years)	0.64	—	1.06	0.73	—	1.04
Gender (male vs. female)	078	—	0.97	0.81	—	0.95
ECOG (0 vs. 1)	0.39	—	0.93	0.35	—	0.91
RTOG-RPA group (III vs. IV-V)	<0.001	<0.001	0.67	<0.001	<0.001	0.63
Symptom duration (<3 vs. ≥3 months)	0.42	—	0.89	0.45	—	0.91
Extent of resection (GTR vs. STR/biopsy)	0.027	0.036	0.53	0.024	0.029	0.48
Anticonvulsant usage (no vs. yes	0.83	—	0.96	0.87	—	0.98
Steroid usage (no vs. yes)	0.17	—	0.84	0.19	—	0.86
Adjuvant TMZ cycles (≤6 vs.7-12)	0.24	—	0.88	0.20	—	0.83
PIV group (L-PIV vs. H-PIV)	<0.001	<0.001	0.38	<0.001	<0.001	0.41

Abbreviations: HR: hazard ratio; ECOG: Eastern Cooperative Oncology Group; RTOG RPA: Radiation Therapy Oncology Group recursive partitioning analysis; GTR: gross total resection; STR: subtotal resection; TMZ: temozolomide; L-PIV: low pan-immune-inflammation value; H-PIV: high pan-immune-inflammation value.

## Data Availability

For researchers who meet the prerequisites for access to sensitive data, the datasets utilized and/or analyzed during the current study are accessible through the Baskent University Department of Radiation Oncology Institutional Data Access (email address: adanabaskent@baskent.edu.tr).
